# The mechanoreceptors in hatchling and adult Elasmobranch skin

**DOI:** 10.17912/micropub.biology.001213

**Published:** 2024-11-19

**Authors:** Angel Amarales, Rebecca Meng, Marco Perez, Michelle Bonilla, Jazmir Hernandez, Maria Elena de Bellard

**Affiliations:** 1 Biology, California State University, Northridge, Northridge, California, United States; 2 Moorpark College, Moorpark, California, United States

## Abstract

The skin is the most extensive organ in vertebrates, composed of two layers: the epidermis and the dermis. Sensory axons originating from the dorsal root ganglia innervate the skin mechanoreceptors in the dermis. Elasmobranchs, which appeared 380 million years ago, are characterized by rough skin composed of dermal denticles. While we know about the epidermis and dermis of elasmobranchs, we do not know much about the presence or abundance of mechanoreceptors in their skin. Using the classic histological hematoxylin and eosin method, we examined the skins of hatchling embryos and adults Batoidea (skates and rays) and Selachimorpha (modern sharks). Our histology findings provide substantial evidence to identify structures with similar morphology to traditional mammalian and reptilian mechanoreceptors like Pacinian and Meissner corpuscles. An interesting observation was the presence of Pacinian in the skin of Batoidea but not in the skin of a Selachimorpha Squalus shark.

**Figure 1. A Hematoxylin & Eosin of elasmobranchs skin.**
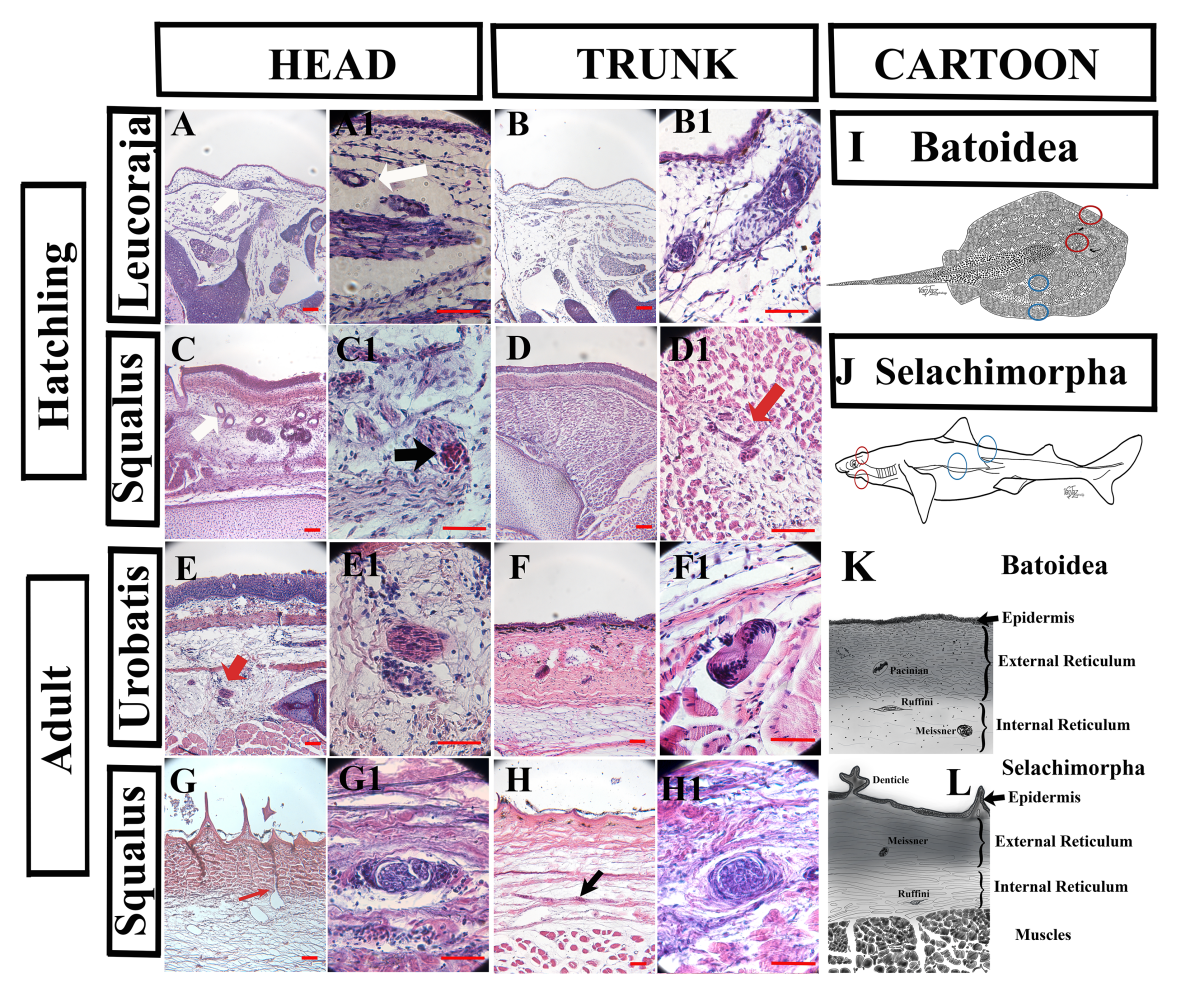
A Hematoxylin & Eosin of elasmobranchs skin. A-D: skin from embryo/hatchling from *Leucoraja* (Batoidea) and *Squalus* (Selachimorpha). A and C show the developing Ampullae de Lorenzini (white arrows). In C1, we observed a developing mechanoreceptor (black arrow). The trunk of both elasmobranchs B1 and D1 (red arrow) shows what might be developing structures. E-H: skin from adult *Urobatis* (Batoidea) and *Squalus* (Selachimorpha). The epidermis and dermis (reticulate layers) are fully developed at this stage, and mechanoreceptors can be distinguished. In *Urobatis* head (E, E1), we observed unknown structures (red arrow). In the *Squalus* head (G, G1), we found Ampullae de Lorenzini ( red arrow) and an encapsulated Ruffini mechanoreceptor (G1). In the trunk of *Urobatis* (F, F1), we identified several Pacinian mechanoreceptors (F1). In the trunk of *Squalus* , we observed several Ruffini mechanoreceptors (black arrow in H) and a Meissner mechanoreceptor (H1). The cartoons summarize the structures we identified (K, L) and the location within the skins (I, J). Red circles correspond to head tissues, and blue to the trunk. K and L cartoons summarize how the elasmobranch skins organize (in the epidermis and reticular layers). We also made cartoons of the mechanoreceptors we observed in their skins. The scalebar dimension for all images is 50 μm.

## Description

The skin is the most extensive organ system of vertebrate bodies. It is composed of two main layers. The outermost layer is the epidermis, derived from ectodermal cells that differentiate into keratinocytes and basal cells. The innermost layer is the dermis, derived from mesodermal cells that populate under the ectoderm. The epidermis primarily protects the organism from infection and injuries, thus evolving as a rough layer in aquatic and land vertebrates (Akat et al., 2022) . The dermis comprises dense or loosely packed dermal fibroblast cells that secrete large amounts of connective tissue, mainly collagen (Hu et al., 2018; McGrath et al., 2004) .

The skin also contains several specialized sensory structures essential to perceiving and responding to environmental changes, making the skin the most significant sensory system in vertebrates (Crowe-Riddell et al., 2019; Roudaut et al., 2012; Soliman SA, 2017; Zimmerman et al., 2014) . Among those specialized structures are mechanoreceptors, which perceive touch, vibration, pressure, and stretch. They are primarily located in the dermis as modified nerve endings (Owens and Lumpkin, 2014) .

Sensory receptors are classified based on the modality to which they are sensitive (mechanoreceptors, thermoreceptors, chemoreceptors, and osmoreceptors), the location of the stimulus to the body (exteroceptors, proprioceptors, and interoceptors), and their morphological basis (capsulated and non-capsulated). In this investigation on elasmobranchs, we intentionally looked for the presence of three encapsulated mechanoreceptors in the epidermis and dermis: Meissner’s corpuscle, Pacinian corpuscle, and Ruffini’s corpuscles, given their clear structures similar to what is known among more recent vertebrates.

Meissner’s corpuscles are tactile receptors made of branched or coiled unmyelinated and myelinated nerve fiber inside a multilayer capsule of flattened Schwann cells form several irregular lamellae and thin connective tissue that is continuous of the endoneurium of the afferent nerve fiber. Their cylindrical shape runs perpendicular to the skin surface and is small (80 um long, 40 um wide). They are tactile receptors distributed just below the epidermis in the skin's connective tissue, and an outer connective tissue separates them from the epidermal layer. (Leeson et al., 1985; Reith and Ross, 1970) .

The Ruffini corpuscle spray-like endings with terminal swellings surrounded by a thin connective tissue capsule. They have elongated fusiform in shape and are made of a single unmyelinated fiber that, upon entering the capsule, forms a dense arborization of delicate axonal endings, each having a small knob-like bulb at their ends. They are mechanoreceptors located in the connective tissue of the dermis (Leeson et al., 1985; Reith and Ross, 1970) .

The Pacinian corpuscles are single, non-myelinated terminal axons encapsulated by concentric layers of modified Schwann and collagenous lamellae made of flattened fibroblast-like cells. The inner layers are more compact than the outer layers, and the layers are filled with fluid. Their unique oval or spherical shape and large size (1–2 mm long and 0.5–1 mm in diameter) make them visible in the skin's dermis at low magnification in histological preparations. They are ubiquitous along species and sensitive to deep pressure and vibratory stimuli in the 20-1000 Hz range (Quindlen-Hotek et al., 2020).

The precise development of skin mechanoreceptors among early vertebrates (Meissner corpuscles, Pacinian corpuscles, and Ruffini) is unknown (Jenkins and Lumpkin, 2017; Owens and Lumpkin, 2014) . The sensory neurons innervating these mechanoreceptors are derived from neural crest cells early in embryogenesis (Zimmerman et al., 2014) . The postnatal development of their specialized encapsulated endings, mainly composed of Schwann cells, is less known (Jenkins and Lumpkin, 2017; Nikolaev et al., 2023; Suazo et al., 2022) .

Elasmobranchs evolved about 380 million years ago (Hara et al., 2018; Yamaguchi et al., 2023) . The skin of elasmobranchs is characterized by an epidermal layer populated by dermal denticles and dermis (or reticular layer), as in other vertebrates (Cooper et al., 2023; Di-Poï and Milinkovitch, 2016). In 2012, Meyer and Seegers's review described the organization of the epidermal and the dermal (reticular) layers of the skin in elasmobranchs (Meyer and Seegers, 2012) . However, this study did not focus on assessing the presence of mechanoreceptors. We examined the skins of hatchling embryos and adults Selachimorpha (modern sharks) and Batoidea (skates and rays) using the classic histological hematoxylin and eosin methods (Fischer et al., 2008) . Our histology findings provide substantial evidence to identify structures with robust analogous morphology to traditional mammalian and reptilian mechanoreceptors. We also include some for which we could not identify their type of sensory organ.

**Elasmobranch Hatchlings** : We first examined the skin in the head and trunk regions of hatchlings *Squalus acanthias* and *Leucoraja erinacea* (Fig.1A-D). In the heads of both *Squalus* and *Leucoraja* , we found the ampullae de Lorenzini (Fig.1A, A1, C, and C1), a unique ancestral electrosensory ampullary organ found in cartilaginous fish, such as sharks, skates, and rays. It is known that the ampullae of Lorenzini and mechanoreceptors have their embryonic origin from the lateral line placodes (Gillis et al., 2012; Modrell et al., 2011; Northcutt et al., 1995) . However, we did not find clear mechanoreceptor structures at this embryonic stage.

The skin of the trunk region of hatchlings from both species contains distinct layers of the impending epidermis and the dermis in adult skin (Fig.1B, B1, D, and D1). In the reticular layer of the dermis of the head, we found scattered condensations of cells, and we propose that they are the precursors of the mechanoreceptors in adult skin. (Fig.1D1)

**Elasmobranch Adults** : Adult tissue, however, shows a different structural makeup. Head : The adult dermal layer of the *Urobatis* and *Squalus* in the head is dense and well-developed. It is characterized by clearly visible denticles in *Squalus* and ampullae de Lorenzini in both species (Modrell and Baker, 2012; O'Neill et al., 2007), reaching from the epidermis into the internal reticular layer. Noticeably, we found *Urobatis* structures close to the internal reticular layer resembling nothing described before (Fig.1E, E1). In the head of *Squalus* , we found Ruffini mechanoreceptors (Fig.1G1), identified based on their capsule. We observed Meissner’s corpuscles in both species. We identified them based on their size and lack of the classic onion-layer structures of the Pacinian/Herbst corpuscle (Soliman SA, 2017) .

Trunk : The trunk region of *Urobatis* shows the presence of the Pacinian/Herbst corpuscles and Meisner mechanoreceptors (Fig.1F, F1). However, in the trunk region of *Squalus* , we did not find any Pacinian/Herbst mechanoreceptors. We noticed small, encapsulated bodies within the reticular layers structurally analogous to Ruffini corpuscles (Soliman SA, 2017) . We also observed structures made of tightly packed cells, as is described for Meisner mechanoreceptors (Suazo et al., 2022) .

Elasmobranch adult skin had well-defined epidermal and dermal/reticular layers and lateral line organs. To illustrate their layers and the mechanoreceptors we observed, we generated two cartoons. One is for batoidea (in our case, *Urobatis* skin), and the other is for selachimorpha (in our case, *Squalus* ). The epidermis of both adult elasmobranchs is thin, with a 3-5-cell layer, while their reticular layers are thicker.

**Conclusions** : This is the first study on mechanoreceptors in the skin of elasmobranchs. Several differences were found in the histological preparations of the adult skin of rays and sharks. A) Ray's internal reticular layer contains an easily distinguishable set of Pacinian corpuscles (Luo et al., 2009) , which are notoriously absent in the skin of the *Squalus* . The lack of these specific mechanical receptors is not unusual in other species; for example, mouse skin does not have Pacinian mechanoreceptors (Handler and Ginty, 2021; Wai et al., 2021) . B) As observed in other mammals, the skin mechanoreceptors in hatchling elasmobranchs still need to develop fully. C) The size of elasmobranchs mechanoreceptors is the same as the ones described in reptiles and mammals. D) Elasmobranch skins lack dermal papillae, which are found in land vertebrates (Bellairs and Osmond, 2014; González-Martínez et al., 2004).

## Methods

**Methods**

A full detailed protocol can be found at ProtocolsIO website: dx.doi.org/10.17504/protocols.io.x54v92m71l3e/v1

**Animal and Tissue Fixation**

*Squalus acanthias* preserved in formalin were purchased from Carolina Biological Supplies, and preserved *Leucoraja* *erinacea* hatchlings from Woods Hole Marine Laboratories were transferred to 4%PFA upon arrival. Live *Urobatis halleri* from Santa Monica Bay were euthanized by tricaine, and the tissues from the head and trunk were collected and placed in 4% PFA.

**Demineralization**

Shark skin from the head (red circles in Fig.1I and J) or trunk (blue circles in Fig.1I, J) was cut into 2x2 cm cube-shaped pieces and submerged in 5M EDTA for demineralization for three days at room temperature (Tomita et al., 2014) .

**Tissue Embedding in Paraffin Sectioning**

After demineralization, tissue samples were submerged in ice-cold 100% methanol overnight in a 50 mL Conical tube. Then, the methanol was decanted and replaced with 200-proof ethanol, which was changed with fresh ethanol twice a day for 3-5 days. Ethanol was replaced with histosol, and when the tissue became partially transparent, we transferred the tissue and histosol into a beaker, warmed them up, added melted paraffin to a 1:1 ratio, and incubated at 59°C overnight. The solution was replaced with fresh paraffin daily to ensure no histosol remained, then embedded in the tissue for the next few days. 10 µm microtome tissue sections were placed on the slide and warmed overnight.

**Hematoxylin and Eosin Staining**

Tissue samples were deparaffinized in xylene and rehydrated in 100% EtOH, 95% EtOH, and DI water. The samples were stained using the standard H&E procedure (described in detail in the ProtocolIO site linked above) and mounted in Permount.
